# Successful treatment of glucocorticoid-resistant acute severe urticaria with JAK1 inhibitor: case report

**DOI:** 10.3389/falgy.2025.1657164

**Published:** 2025-09-19

**Authors:** Ying Wu, Long-fei Wang, He-nian Yang, Chen-xing Kan, Xuan Guo, Guo-dong Hao

**Affiliations:** Department of Allergy, Tangshan Workers Hospital, Tangshan, Hebei, China

**Keywords:** acute severe urticaria, JAK1 inhibitors, upadacitinib, glucocorticoid-resistant, successful treatment

## Abstract

**Objective:**

This study analyzes a case with a JAK1 (Janus Kinase 1) inhibitor was successfully employed to treat a patient with glucocorticoid-resistant acute severe urticaria (ASU), with the aim of improving clinical understanding of this condition.

**Methods:**

A retrospective analysis was conducted on the clinical data, diagnosis, treatment, and prognosis of a patient with acute severe urticaria, who was admitted to the Allergy Department of Tangshan Workers’ Hospital on March 10, 2025.

**Results:**

The patient was a 50-year-old female who presented with widespread skin wheals and itching, along with a sensation of throat obstruction for two days. Upon admission, the patient had a body temperature of 38.5°C. Large, irregularly shaped wheals, up to 10 cm in diameter, were observed on the skin. These wheals were bright red with surrounding erythema and increased upon scratching. Laboratory tests indicated elevated levels of white blood cells (WBC), neutrophils percentage, neutrophils absolute value, total IgE, and interleukin-6 (IL-6). A diagnosis of acute severe urticaria was made. Prior to admission, the patient had been administered with betamethasone sodium phosphate, dexamethasone sodium phosphate, methylprednisolone succinate, diphenhydramine, and calcium gluconate at the emergency department without relief in wheals and itching. Upon admission, the patient was treated with glucocorticoids and JAK1 inhibitors, resulting in the complete regression of the rash and normalization of laboratory indicators.

**Conclusion:**

This case suggests that JAK1 inhibitors can achieve satisfactory results in treating glucocorticoid-resistant acute severe urticaria.

## 1 Introduction

Acute spontaneous urticaria is defined as the occurrence of spontaneous wheals, angioedema or both for less than 6 weeks (1). Acute urticaria often presents with multi-system symptoms, including respiratory and digestive manifestations such as nausea, vomiting, abdominal pain, diarrhea, chest tightness, and throat obstruction (2). These patients exhibit a Th2 cell-dominated T-helper cell imbalance (3). Th2 cells play roles in allergic diseases, not limited to promoting B-cell IgE production, but also in generating cytokines like IL-4, 5, and 13 (4). These cytokines signal via the JAK-STAT pathway, mediating inflammation and itching. Over 50 cytokines have been identified as signaling through the JAK-STAT pathway, with JAK receptors possibly linked to multiple cytokine receptors (5). By inhibiting JAK1 kinase activation, JAK1 inhibitors block cytokine signaling, providing rapid anti-inflammatory and anti-itch action, notably in treating refractory or immune-mediated inflammatory diseases. To date, no cases have been reported on the use of JAK1 inhibitors for glucocorticoid-resistant acute severe urticaria. This article presents such a case to enhance understanding of this treatment.

## 2 Clinical data

### 2.1 General information

The patient, a 50-year-old female, was admitted for widespread skin wheals with severe itching and throat obstruction sensation on March 10, 2025. The onset was March 8, marked by large skin wheals, intolerable itching, and persistent symptoms not relieved by self-care. There was no throat pain, cough, sputum, abdominal pain, diarrhea, nausea, vomiting, urinary issues, or joint pain. The wheals were unrelated to cold or heat stimuli. Previously treated at the emergency room with intravenous medications such as betamethasone sodium phosphate, dexamethasone sodium phosphate, methylprednisolone succinate, diphenhydramine, and calcium gluconate with minimal relief from throat obstruction and persistent wheals and itching. No past medical history of hepatitis, tuberculosis, or other infectious diseases was reported. The patient denied any drug or food allergies, with no family history of similar illnesses.

### 2.2 Physical examination and auxiliary tests

**Figure 1:**
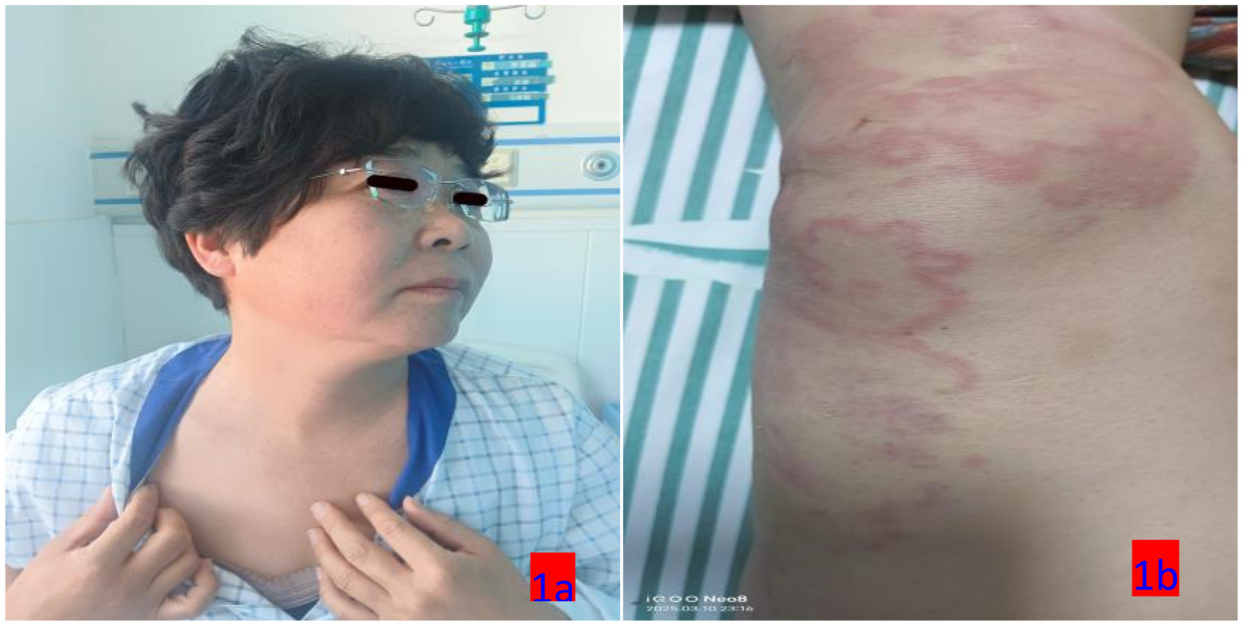
**(a,b)** The condition of urticaria on the first day of admission (March 10, 2025).

Upon admission: mental status was poor, consciousness clear, temperature 37.9°C, pulse 96 beats/min, respiratory rate 20 breaths/min, blood pressure 110/75 mmHg. The patient displayed large skin wheals, up to 10 cm, irregularly shaped, bright red with surrounding erythema, as shown in Figures 1a,b. Elevated skin temperature, absence of sweating or joint deformation, and no swollen lymph nodes were noted. The Urticaria Activity Score (UAS7) (6) was 36, and the Chronic Urticaria Quality of Life Questionnaire (CU-Q2oL) (7) scored 35. Auxiliary tests on the admission day are shown in Table 1.

**Table 1:** List of auxiliary examination results of the patient on the day of admission.

Blood project	Measured value	Reference value	Blood project	Measured value	Reference value
White blood cell (WBC)	17.21 × 10^9^/L	3.5–9.5 × 10^9^/L	Antinuclear antibody combination	Negative (−)	<1: 80
neutrophils absolute value (NEUT#)	16.54 × 10^9^/L	1.8–6.3 × 10^9^/L	Combination of autoantibodies	Negative (−)	Negative −<15
Lymphocytes absolute value (LYMPH#)	1.5 × 10^9^/L	1.1–3.2 × 10^9^/L	Complement C3 (C3)	1.45	0.78–2.1
Neutrophils percentage (NEUT%)	0.96	0.4–0.75	Complement C4 (C4)	0.24	0.17–0.48
Lymphocytes percentage (LYMPH%)	0.30	0.2–0.5	Interleukin-2 (IL-2)	<2.44	≤7.5 pg/ml
Eosinophils absolute value (EO#)	0.03	0.02–0.52 × 10^9^/L	Interleukin-4 (IL-4)	<2.44	≤8.56 pg/ml
Eosinophils percentage (EO%)	0.00	0.004–0.08	Interleukin-5 (IL-5)	3.04	≤3.1 pg/ml
Procalcitonin (PCT)	<0.05	0–0.05 ng/ml	Interleukin-6 (IL-6)	35.73	≤5.4 pg/ml
Erythrocyte sedimentation rate (ESR)	23.00	0–25 mm/h	Interleukin-8 (IL-8)	15.8	≤20.6 pg/ml
Tota Immunoglobulin E (TIgE)	155.20	0–100 IU/ml	Interleukin-10 (IL-10)	<2.44	≤12.9 pg/ml
reactive protein (CRP)	5.6	0–10 mg/L	Interleukin-17 (IL-17)	<2.44	≤21.4 pg/ml
Antistreptolysin O (ASO)	134.20	0–160 IU/ml	Interleukin-12P70 (IL-12P70)	<2.44	≤3.4 pg/ml
Respiratory syncytial virus (RSV)	Negative (−)	Negative (−)	Interleukin-1β (IL-1β)	2.48	≤12.4 pg/ml
RNA viruses of influenza A and B (FluA, FluB)	Negative (−)	Negative (−)	Interferon-a (IFN-a)	<2.44	≤8.5 pg/ml
Human rhinovirus RNA (HRV)	Negative (−)	Negative (−)	Interferon-*γ*(IFN-γ)	6.81	≤23.1 pg/ml
Adenovirus (ADV)	Negative (−)	Negative (−)	Tumor necrosis factor-a (TNF-a)	<2.44	≤16.5 pg/ml
Mycoplasma pneumoniae (MP)	Negative (−)	Negative (−)	Serum immunoglobulin E(sIgE)	Negative (−)	0≤sIgE<0.35

### 2.3 Diagnosis and treatment process

**Figure 2:**
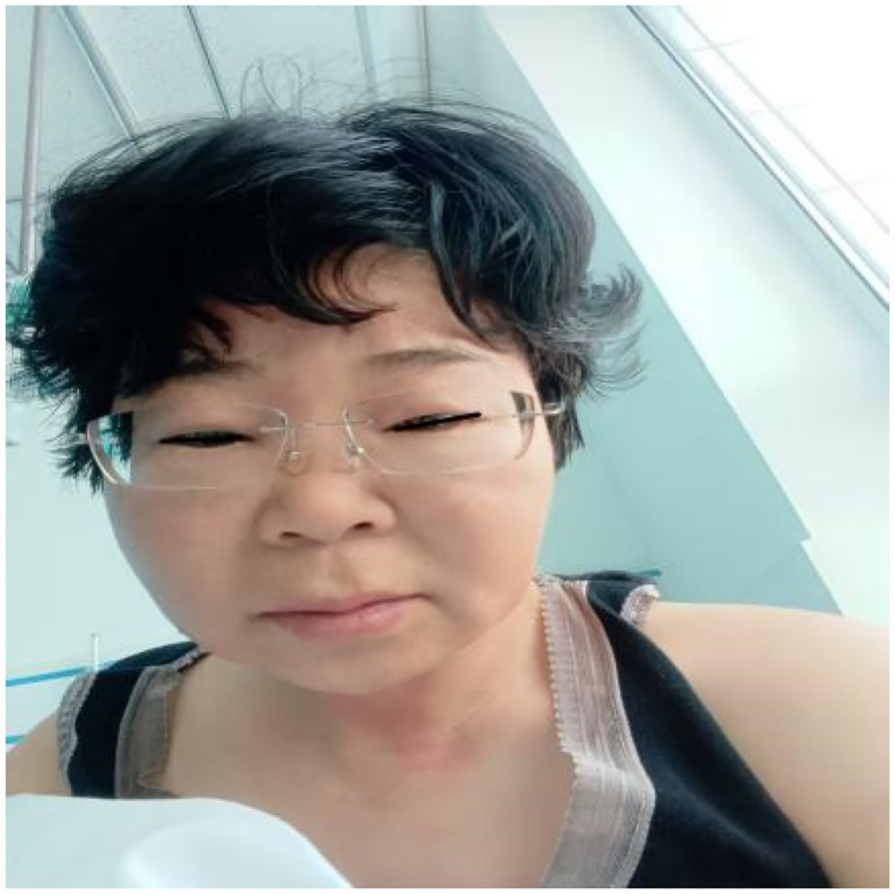
On the second day of hospitalization (March 11, 2025), the patient exhibited swelling in the eyelids and face, along with a rash on the neck.

**Figure 3:**
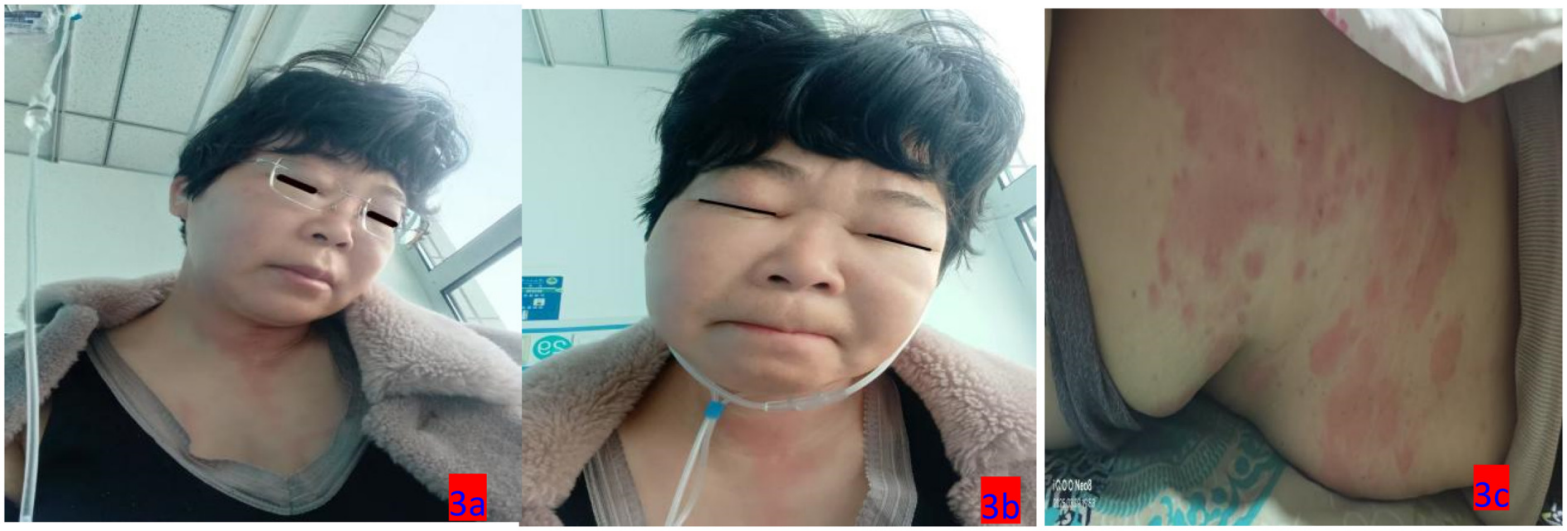
**(a–c)** On the third day of hospitalization (March 12, 2025), the patient exhibited swelling in the eyelids and face, along with truncal rashes.

**Figure 4:**
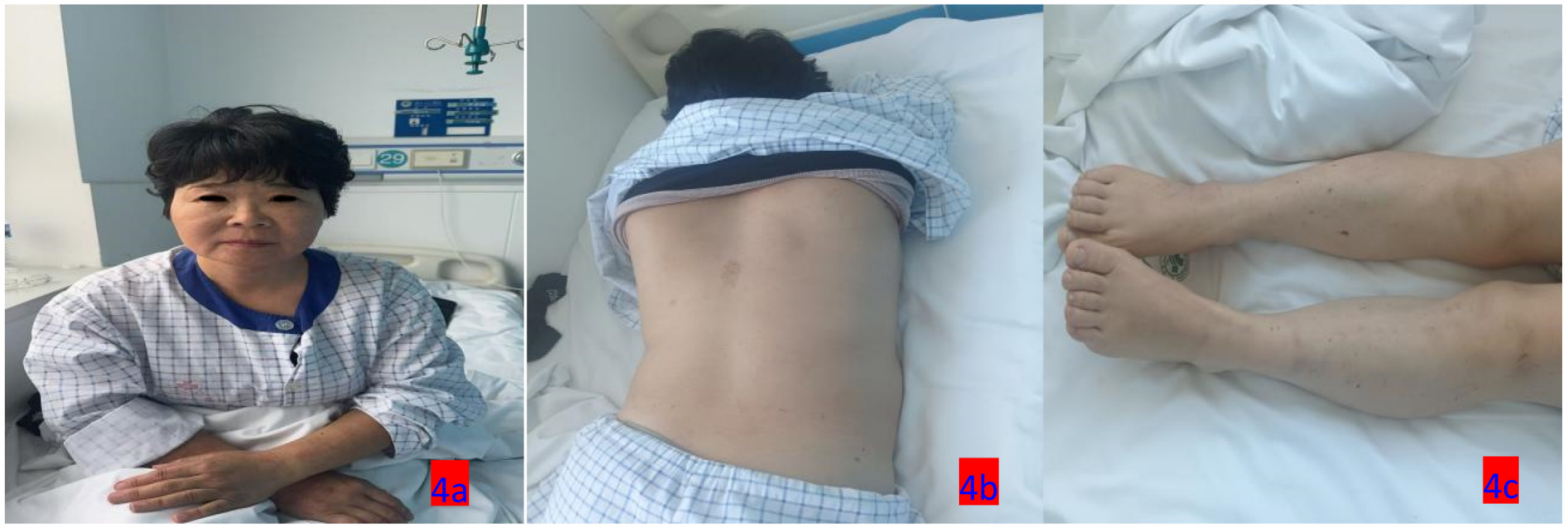
**(a–c)** On the sixth day of hospitalization (March 16, 2025), the patient exhibited a reduction in eyelid and facial rashes, as well as improvement in skin lesions throughout the body.

**Figure 5:**
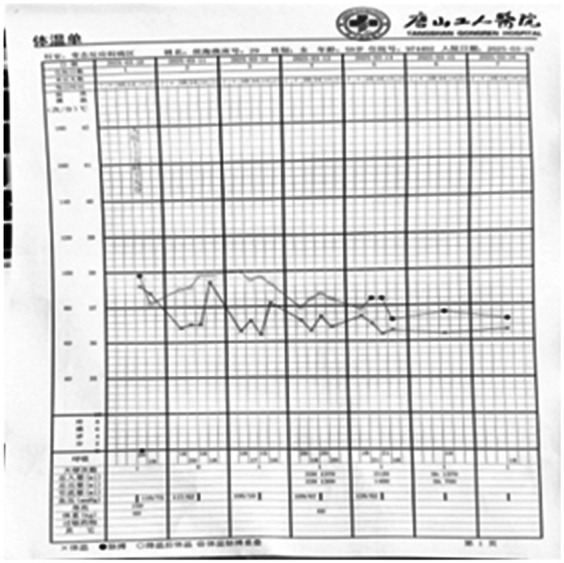
Illustrates the changes in the patient's vital signs during hospitalization.

Following comprehensive evaluation and exclusion of contraindications, the diagnosis of acute severe urticaria was confirmed. Details of diagnosis and treatment are listed in Table 2 (Figures 2–5).

**Table 2:** Treatment process of patients before and after admission.

Date	Symptoms	Signs	Treatment	See the picture
3.8	Severe itching is accompanied by skin wheals that do not spontaneously resolve, along with a sensation of throat obstruction and fever.	T: 38.5℃ (Figure 5), The patient exhibited extensive skin wheals	Intravenous administration included betamethasone sodium phosphate (8 mg), dexamethasone sodium phosphate (5 mg), methylprednisolone sodium succinate (40 mg), diphenhydramine hydrochloride (20 mg), and calcium gluconate (10 mg).	None
3.10	The patient presented with widespread skin wheals accompanied by severe itching that did not resolve spontaneously, along with a sensation of throat obstruction	T: 37.9℃ (Figure 5), The skin wheals had diameters of up to 10 cm and displayed irregular shapes. Some were isolated, while others merged into clusters. The wheals were bright red with surrounding erythema UAS7 score: 36分 CU-Q2oL score: 35分	Intravenous administration included methylprednisolone sodium succinate (80 mg every 12 h) and calcium gluconate (20 ml once daily). Budesonide suspension was administered via nebulization (1 mg twice daily). Additionally, furosemide (10 mg) was administered intravenously. Oral medications included ebastine tablets (10 mg once daily) and loratadine tablets (10 mg once daily).	Figure 1
3.11	There was no reduction in the skin wheals, and eyelid edema developed	T: 37.7℃ (Figure 5), Extensive skin wheals were observed, with slight eyelid edema.	Same as 3.10	Figure 2
3.12	The number of skin wheals increased, with worsening throat obstruction and enhanced facial and perioral edema	T: 37.1℃ (Figure 5), The skin wheals persisted, accompanied by worsening eyelid edema and perioral swelling	Sanme as 3.10–3.11	Figures 3a–c
3.13	The skin wheals reduced in number, itching was significantly alleviated, and throat obstruction improved	T: 36.7℃ (Figure 5), There was a reduction in wheals, as well as eyelid and perioral edema.	Sanme as 3.10–3.11 Added Upadacitinib 30mg PO daily	None
3.14–3.15	The patient's skin wheals gradually disappeared	After three days of treatment, the patient's skin wheals disappeared, with no eyelid or perioral edema present	Betamethasone sodium phosphate was administered intravenously at a dosage of 12 mg once daily. Calcium gluconate was also given intravenously at 20 ml once daily. Upadacitinib extended-release tablets were taken orally at a dose of 30 mg once daily.	Figures 4a–c
3.16–3.17	The patient's skin wheals completely resolved.	No skin wheals or edema were detected	Below is a paragraph from an academic paper. Polish the writing to meet the acdemic style, improve the spelling, grammar, clarity, concision and overall readability. When necessary, rewrite the whole sentence. Furthermore, list all modification and explain the reason to do so in markdown table.	None
3.18–3.19	The patient's skin wheals completely resolved.	The absence of skin wheals and edema was confirmed. UAS7 score: 5 CU-Q2oL score: 8	Betamethasone sodium phosphate was administered intravenously at a dose of 4 mg once daily. Calcium gluconate was also administered intravenously, with a dosage of 20 ml once daily. Upadacitinib extended-release tablets were taken orally at a dose of 30 mg once daily.	None
Prognosis and follow-up	The patient's condition stabilized with no facial redness, disappearance of skin wheals, and no new lesions	The patient maintained a normal body temperature with no skin wheals or edema	After discharge, the patient continued to take Upadacitinib extended-release tablets at a dose of 30 mg once daily for six days, along with Ebastine at a dose of 10 mg once daily for six days.	None

## 3 Discussion

The primary etiologies of acute urticaria are infections, medications, and foods (8). Infections are closely related to its onset, with bacterial, fungal, viral, and parasitic factors (9). Acute infectious urticaria presents with fever, throat pain, joint pain, chest tightness, and abdominal pain; lab tests frequently show elevated inflammatory markers. A Japanese study set the following diagnostic criteria for acute infectious urticaria: widespread wheals with fever (≥37°C), resistance to antihistamine treatment requiring glucocorticoid and antibiotic combinations, and meeting two of three lab abnormalities (WBC >10,000/mm^3^, neutrophils percentage >70%, CRP >0.5 mg/dl) (10). In this case, the patient's widespread, intractable wheals were bright red and merged into larger patches, causing intense itching. Glucocorticoid treatment saw no symptom relief but induced fever, worsening throat obstruction, eyelid, and facial edema. No infection was observed clinically or in lab tests except elevated WBC, neutrophils, and IL-6. The presentation aligns with infectious urticaria diagnostic criteria, though wheals preceded fever with a low fever magnitude suggesting possible severe allergy or nosocomial infection. Literature shows increased white blood cells and neutrophils in patients treated with glucocorticoids (11), making symptom and lab tests insufficient for classifying infectious urticaria. Without comprehensive differentiation between infectious/non-infectious urticaria, symptoms resolved rapidly with Upadacitinib, highlighting its importance in early infectious/non-infectious urticaria treatment.

Upadacitinib is a selective JAK1 inhibitor affecting CD4+ T cells, neutrophils, dendritic cells, and reducing inflammatory cytokines like IL-6, IL-17, IL-2, IL-23, IL-36, IFN-α, IFN-β, and IFN-γ (12), influencing Th2 cell differentiation and inflammation cell infiltration, thereby reducing mast cell activation, inflammatory mediator release, and alleviating urticaria symptoms. Upadacitinib not only inhibits inflammatory pathways but also modulates immune cell functions, aiding immune balance recovery. This dual regulatory role aids in controlling urticaria's inflammatory state.

In summary, this case reports the successful use of the JAK1 inhibitor Upadacitinib for glucocorticoid-resistant acute severe urticaria, achieving satisfactory results. JAK1 inhibitors could become highly promising treatment options for acute severe urticaria patients. No thorough differentiation was made for infectious urticaria in this case; thus, no anti-infective treatment was administered. Further studies on the long-term efficacy and safety of JAK1 inhibitors in acute and chronic severe urticaria are warranted.

## Data Availability

The original contributions presented in the study are included in the article/Supplementary Material, further inquiries can be directed to the corresponding author.
